# Association between dietary index for gut microbiota and female infertility: a cross-sectional NHANES 2013–2020

**DOI:** 10.3389/fnut.2025.1587240

**Published:** 2025-04-23

**Authors:** Yu Fu, Mengling Peng, He Cai, Bing Li, Yaoting Zhang, Yang Zheng

**Affiliations:** Department of Cardiovascular Diseases, The First Hospital of Jilin University, Changchun, China

**Keywords:** dietary index for gut microbiota, infertility, body mass index, mediation analysis, NHANES

## Abstract

**Background:**

The relationship between the gut microbiota and infertility has garnered increasing attention. However, the associations between dietary index for gut microbiota (DI-GM), an indicator of microbial diversity, and infertility remain insufficiently explored.

**Methods:**

We analyzed data from 3,058 participants in the NHANES 2013–2020 cycles, employing weighted generalized linear models and smooth curve analyses to examine their associations. Mediation analysis was conducted to assess the role of body mass index (BMI).

**Results:**

After adjusting for confounding factors, a higher DI-GM score was significantly associated with a lower prevalence of infertility (OR = 0.89, 95% CI = 0.81–0.98, *p* = 0.029). Compared with individuals with a DI-GM score of 0–3, those with a score ≥6 presented a significantly lower prevalence of infertility (OR = 0.64, 95% CI = 0.43–0.96, *p* = 0.039). BMI mediated 5.98% of the association between DI-GM and infertility.

**Conclusion:**

A higher DI-GM score is associated with a lower prevalence of infertility. Future studies should employ longitudinal designs to validate these findings.

## 1 Introduction

Globally, the incidence of female infertility is increasing significantly, affecting younger populations. Epidemiological studies indicate that this condition affects millions of women of reproductive age (1). The World Health Organization (WHO) has warned that infertility is emerging as the third major public health challenge, following malignant tumors and cardiovascular diseases. In the United States, approximately 12.7% of women of reproductive age require medical intervention for infertility each year (2). Despite advancements in assisted reproductive technologies, the complexity of infertility and its substantial socioeconomic burden (3, 4) underscore the urgent need for novel preventive and therapeutic strategies.

Recent studies suggest that dietary interventions play a crucial role in the prevention and management of infertility. A prospective cohort study revealed that optimizing dietary patterns significantly increased live birth rates (5). The underlying mechanisms may operate through two primary pathways: first, dietary components directly influence reproductive endocrine homeostasis; second, as key environmental factors shaping the gut microbiota (6), dietary patterns modulate microbial composition, thereby affecting host metabolism. Notably, the symbiotic relationship between the gut microbiota and the host comprises more than 100 trillion microbial cells. These microorganisms secrete *β*-glucuronidase, which modulates the enterohepatic circulation of estrogen, thereby influencing reproductive function (7). When dietary imbalances lead to gut dysbiosis, the resulting decline in microbial diversity significantly reduces *β*-glucuronidase activity, leading to decreased circulating estrogen levels and metabolic disturbances, ultimately impairing fertility (8). To quantify the dietary–microbiota interaction, researchers developed dietary index for gut microbiota (DI-GM) on the basis of a systematic review of 106 studies. This DI-GM employs a weighted scoring system for 14 food groups/nutrients, providing a comprehensive assessment of the impact of diet on microbial composition and diversity (9). Compared with traditional invasive testing methods, DI-GM enables large-scale population assessments through non-invasive dietary surveys, offering unique advantages in epidemiological research platforms. In particular, the National Health and Nutrition Examination Survey (NHANES) provides an ideal data source for applying the DI-GM due to its nationally representative sample, standardized 24-h dietary recall method, and rich demographic, clinical, and laboratory data. These features enable robust, population-level analyses of the association between diet–microbiota interactions and infertility while allowing for adequate control of potential confounding factors.

The DI-GM has been demonstrated to be significantly inversely associated with the risk of stroke and diabetes (10, 11); however, its association with infertility remains unexplored. Moreover, given that obesity, as assessed by body mass index (BMI), is a major risk factor for infertility (7) and that gut microbiota-derived metabolites (e.g., butyrate) can regulate lipid metabolism (7), investigating the mediating role of BMI in the association between the DI-GM and infertility holds significant scientific value.

## 2 Methods

### 2.1 Study

This study utilized data from the NHANES 2013–2020 cycles. The NHANES is an ongoing cross-sectional survey conducted on the non-institutionalized U.S. population that employs a multistage probability sampling method to collect health, nutritional, and demographic data from participants. The data used in this study were derived from publicly available files and were approved by the National Center for Health Statistics (NCHS) Ethics Review Board. All participants provided written informed consent. This study adhered to the Strengthening the Reporting of Observational Studies in Epidemiology (STROBE) guidelines.

### 2.2 Study design and population

**Figure 1:**
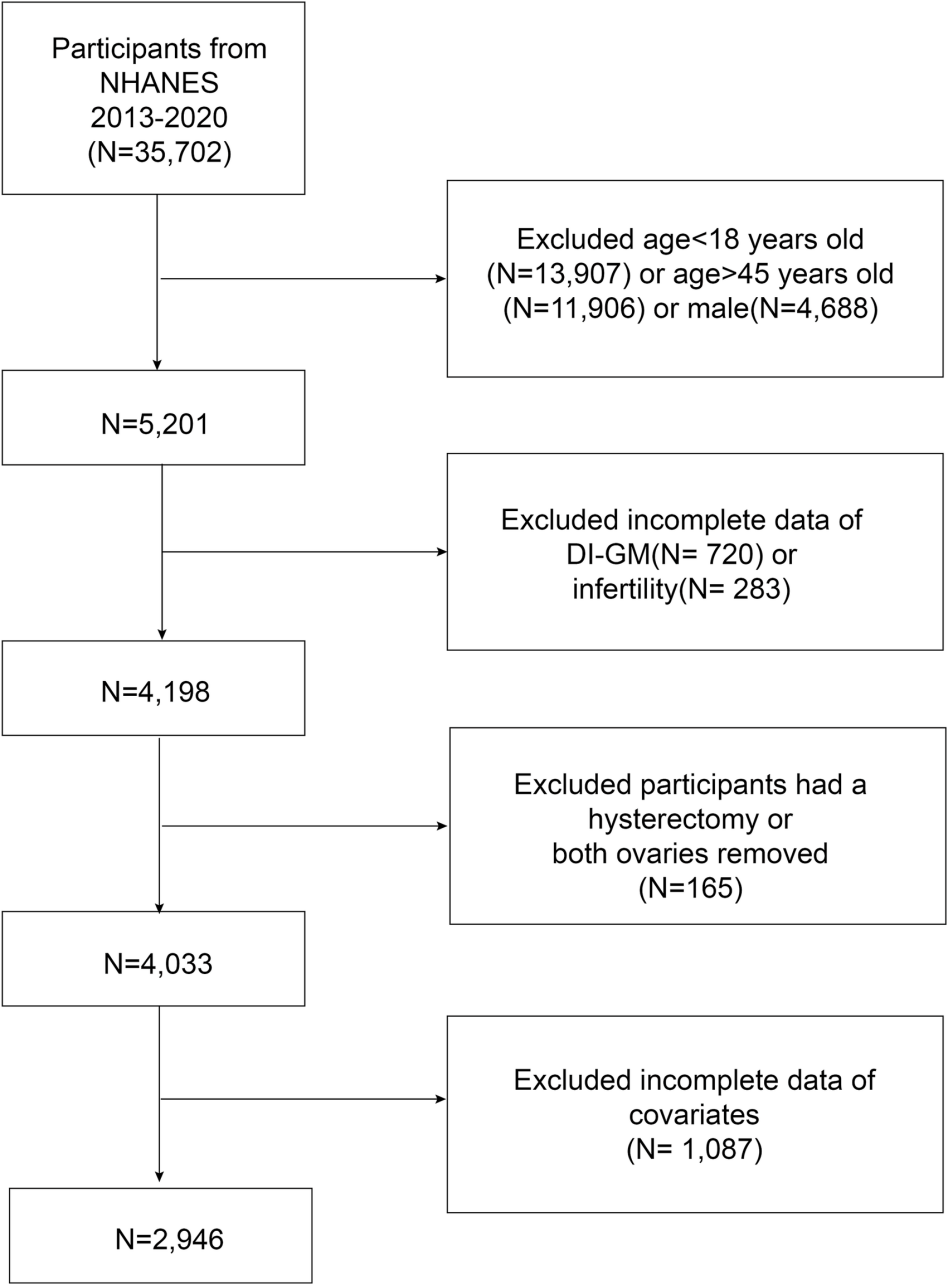
Flow chart.

The participants included women of reproductive age (18–45 years) who participated in the NHANES from 2013 to 2020. Individuals with missing data on DI-GM components or infertility diagnoses were excluded. Additionally, women who had undergone hysterectomy or bilateral oophorectomy were excluded because they may not have attempted pregnancy. Finally, participants with missing data on covariates were excluded from the analysis. A total of 2,946 eligible participants were included in the analysis, of whom 406 were diagnosed with infertility (Figure 1).

### 2.3 DI-GM

The DI-GM consists of 14 foods or nutrients, with beneficial components, including fermented dairy, chickpeas, soybean, whole grains, fiber, cranberries, avocados, broccoli, coffee, and green tea, and unfavorable components, including red meat, processed meat, refined grains, and a high-fat diet (≥40% of energy from fat) (9). The DI-GM score was calculated via 24-h dietary recall data from the NHANES. For beneficial components, a score of 1 was assigned if consumption was ≥ the sex-specific median; otherwise, a score of 0 was assigned. These scores were summed to yield the beneficial-to-gut microbiota score (BGMS, ranging from 0 to 10). For unfavorable components, a score of 0 was assigned if consumption was ≥ the sex-specific median or 40% (for a high-fat diet); otherwise, a score of 1 resulted in an unfavorable gut microbiota score (UGMS, ranging from 0 to 4). The DI-GM score (ranging from 0 to 14) was obtained by summing these component scores and categorized into four groups: 0–3, 4, 5, and ≥6 (10). The components and scoring criteria of DI-GM are detailed in Supplementary Table S1.

### 2.4 Infertility

In accordance with the definition of infertility (2), women who answered “yes” to either of the following questions were classified as having experienced infertility: “Have you ever tried to get pregnant for at least 1 year without success?” or “Have you ever consulted a doctor or other healthcare provider due to difficulty conceiving?”.

### 2.5 Covariates

The selection of covariates in this study was based on expert judgment and previous research (2, 12). The included covariates included age, race (non-Hispanic Black, non-Hispanic White, Mexican American, or other races), education level (less than high school, high school or equivalent, college or above) (10), the ratio of family income to the poverty line [low income (≤1.3), middle income (>1.3–3.5), and high income (>3.5)] (13), marital status (married, never married, living with partner, other) (10), smoking status (have you smoked at least 100 cigarettes in your entire life?) (14), alcohol consumption (frequency of drinking more than 12 times in the past year) (13), physical activity (participants were considered physically active if they engaged in moderate/vigorous work/recreational activities) (15), body mass index (BMI, weight divided by height squared) (16), age at menarche, menstrual regularity, history of pelvic infection, and history of female hormone use, depression was assessed via the Patient Health Questionnaire-9 (PHQ-9), a validated instrument based on DSM-IV criteria, with a total score ≥10 considered indicative of clinically significant depressive symptoms. This cutoff has demonstrated strong diagnostic performance, with a sensitivity and specificity of 88% (17). Hypertension was defined as meeting any of the following criteria: current use of antihypertensive medication, self-reported diagnosis on at least two occasions by a physician, or an average systolic blood pressure ≥140 mmHg and/or diastolic blood pressure ≥90 mmHg on the basis of three consecutive measurements (18, 19). Hyperlipidemia was defined according to the National Cholesterol Education Program Adult Treatment Panel III criteria as having total cholesterol ≥200 mg/dL, triglycerides ≥150 mg/dL, LDL-C ≥ 130 mg/dL, HDL-C ≤ 50 mg/dL (for women), or current use of lipid-lowering medication (20). Diabetes was defined as a self-reported physician diagnosis, HbA1c ≥ 6.5%, fasting blood glucose ≥126 mg/dL, or the use of insulin or oral hypoglycemic agents (21). These covariates were obtained from the demographic, dietary, examination, laboratory, and questionnaire sections of the NHANES database.

### 2.6 Statistical analysis

Statistical analyses were conducted via R (version 4.4.1) and EmpowerStats (version 4.2). Since the NHANES employs a stratified, multistage, probability sampling design, the “survey” package was utilized for analysis. The baseline characteristics of the NHANES study population were statistically described according to infertility status. For continuous variables, the means ± standard deviations were calculated via survey-weighted linear regression (svyglm). Survey-weighted percentages for categorical variables were obtained via survey-weighted linear regression (svyglm). Weighted multivariate generalized linear regression was employed to calculate odds ratios (ORs) and 95% confidence intervals (CIs) for the association between DI-GM and infertility. Three models were employed for analysis: Model 1: Crude model without adjustment for any covariates. Model 2: Adjusted for age and race. Model 3: Model 3 was further adjusted for age, race, education level, PIR, BMI, marital status, smoking status, alcohol intake, age at menarche, pelvic infection, ever use of female hormones, menstrual regularity, physical activity, depression, hypertension, diabetes, and hyperlipidemia. Smooth curve fitting was subsequently performed to further assess the relationship between DI-GM and infertility.

The sensitivity analyses included the following: (1) Subgroup analysis. (2) Multiple imputation: Missing data were handled via the multiple imputation by chained equations (MICE) method, generating five imputed datasets. Further details on multiple imputation are provided in the Supplementary methods. (3) Analysis using unweighted data.

The mediation package in R was used to conduct 1,000 bootstrap simulations to estimate the 95% confidence interval for the mediation effect (22), assessing the mediating role of BMI in the association between DI-GM and infertility. Receiver operating characteristic (ROC) curves were used to evaluate the predictive efficacy of the nomogram incorporating the DI-GM and other covariates for infertility. The predictive performance was assessed via the area under the ROC curve (AUC).

## 3 Results

### 3.1 Participant characteristics

As shown in Table 1, this study included 2,946 participants from the 2013–2020 NHANES dataset, of whom 406 were diagnosed with infertility, whereas 2,540 did not have infertility. The mean age of the participants was 32.29 years (SD = 7.49). Compared with non-infertile individuals, those with infertility were older (34.57 years vs. 31.93 years, *p* < 0.001). Significant differences were also observed in PIR, marital status, smoking status, BMI, history of pelvic infection, history of female hormone use, diabetes, depression, hyperlipidemia and hypertension (*p* < 0.05).

**Table 1:** Characteristics of the NHANES 2013–2020 participants.

Characteristic	Infertility		*p*-value
	Negative (*N* = 2,540)	Positive (*N* = 406)	
Age, mean ± SD	31.93 ± 7.52	34.57 ± 6.90	**<0.001**
Age, %			**<0.001**
18–30	48.40	27.49	
30–45	51.60	72.51	
Race, %			0.657
Mexican American	11.48	11.30	
Other Hispanic	7.88	5.89	
Non-Hispanic White	57.25	60.69	
Non-Hispanic Black	13.37	13.51	
Other Race	10.02	8.61	
Education level, %			0.813
Less than high school	9.51	10.80	
High school or equivalent	19.11	18.75	
College or above	71.38	70.45	
Marital status, %			**<0.001**
Married	48.95	68.51	
Never married	18.57	5.76	
Living with partner	9.08	5.32	
Other	23.39	20.41	
PIR, %			**0.014**
≤1.3	28.83	20.29	
1.3–3.5	35.77	41.89	
>3.5	35.40	37.82	
Smoking status, %			**0.026**
No	68.57	60.40	
Yes	31.43	39.60	
Alcohol intake, %			0.987
No	45.93	46.00	
Yes	54.07	54.00	
Physical activity, %			0.099
Inactive	20.95	25.76	
Active	79.05	74.24	
Age at Menarche, mean ± SD	12.52 ± 1.76	12.41 ± 1.87	0.483
Pelvic infection, %			**0.001**
No	96.54	89.36	
Yes	3.46	10.64	
Ever use female hormones, %			**0.034**
No	96.87	92.99	
Yes	3.13	7.01	
Menstrual regularity, %			0.127
No	5.94	8.81	
Yes	94.06	91。19	
DI-GM score, Mean ± SD	4.48 ± 1.56	4.31 ± 1.60	**0.029**
DI-GM group, %			0.239
0–3	24.81	30.43	
4	24.99	25.43	
5	22.92	22.21	
≥6	27.28	31.82	
Beneficial to gut microbiota	1.90 ± 1.28	1.89 ± 1.32	0.151
Unfavorable to gut microbiota	2.58 ± 1.05	2.40 ± 1.08	0.104
BMI (kg/m^2^), mean ± SD	29.63 ± 8.46	32.23 ± 9.40	**0.003**
BMI, %			**0.001**
≤25	38.49	28.16	
25–30	24.56	20.49	
>30	36.95	51.35	
Depression, %			**0.001**
No	92.50	85.09	
Yes	7.50	14.91	
Hypertension, %			**0.010**
No	89.65	84.24	
Yes	10.35	15.76	
Hyperlipidemia, %			**0.013**
No	48.62	37.06	
Yes	51.38	62.94	
Diabetes, %			**<0.001**
No	95.91	90.17	
Yes	4.09	9.83	

Mean ± standard deviation for continuous variables: *P*-value was calculated by survey-weighted linear regression (svyglm). Survey-weighted percentages for categorical variables: survey-weighted linear regression (svyglm). BMI, body mass index; DI-GM, dietary index for gut microbiota; PIR, poverty income ratio. The bold values in Table 1 indicate *p* < 0.05.

### 3.2 Relationship between DI-GM and infertility

**Figure 2:**
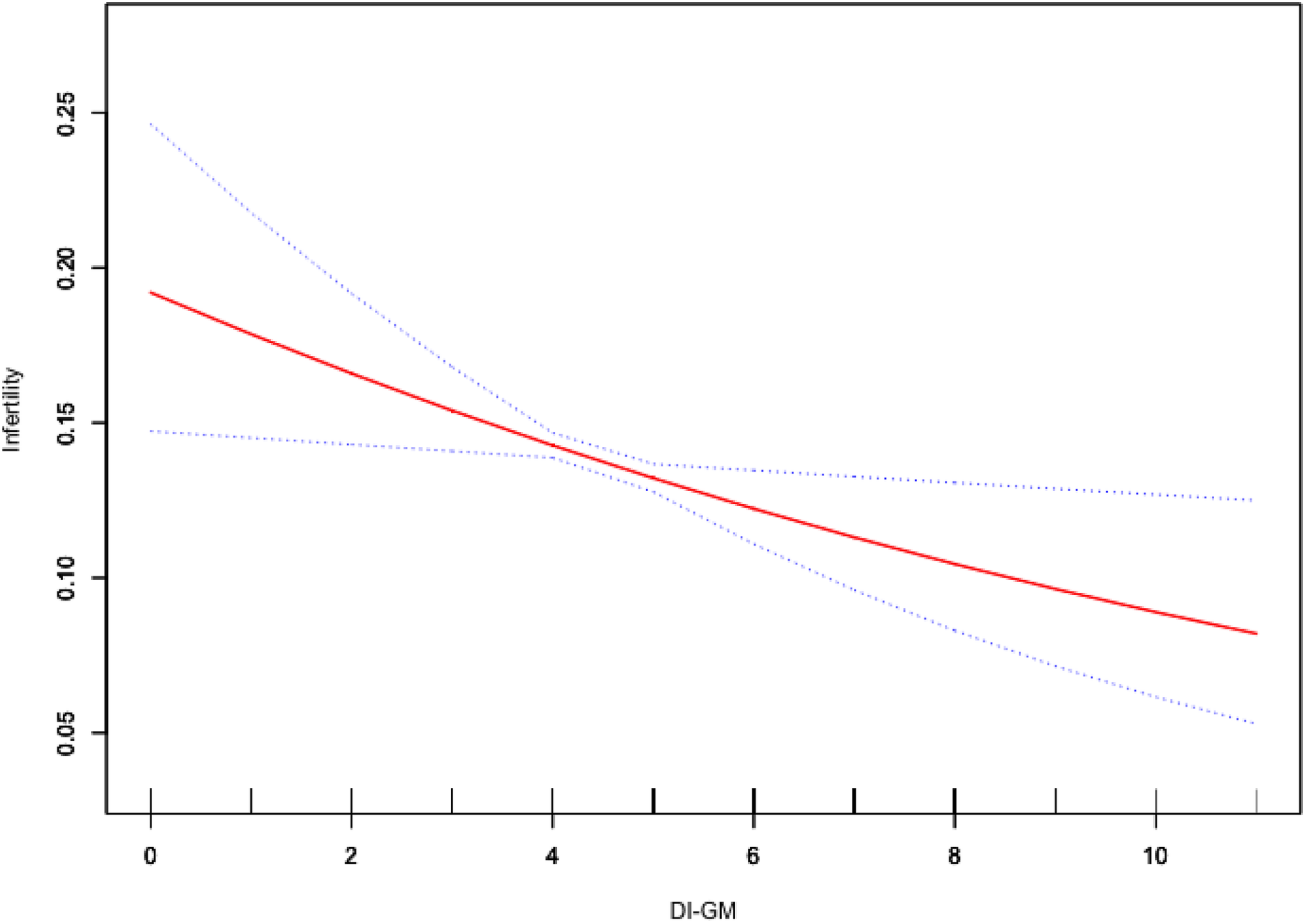
Association between DI-GM and infertility. Age, race, education level, PIR, BMI, marital status, smoking status, alcohol intake, age at menarche, pelvic infection, ever use of female hormones, menstrual regularity, physical activity, depression, hypertension, diabetes, and hyperlipidemia were adjusted. BMI, body mass index; DI-GM, dietary index for gut microbiota; PIR, poverty income ratio.

As shown in Table 2, weighted generalized linear model analysis indicated that higher DI-GM scores were associated with a lower prevalence of infertility. In Model 3, for each one-unit increase in DI-GM, the odds of infertility decreased by 11% (OR = 0.89, 95% CI = 0.81–0.98, *p* = 0.029). Compared with the group with a DI-GM score of 0–3, patients with a score ≥6 (OR = 0.64, 95% CI = 0.43–0.96, *p* = 0.039) had a significantly lower prevalence of infertility. The smooth curve fitting analysis further illustrated the inverse association between DI-GM scores and infertility (Figure 2).

**Table 2:** Association between DI-GM and infertility in NHANES.

Variable	Model 1	Model 2	Model 3
	OR (95% CI) *p*-value	OR (95% CI) *P*-value	OR (95% CI) *P*-value
DI-GM	0.89 (0.81, 0.98) 0.023	0.88 (0.79, 0.97) 0.014	0.89 (0.81, 0.98) 0.029
DI-GM group			
0–3	Reference	Reference	Reference
4	0.83 (0.56, 1.21) 0.332	0.79 (0.54, 1.16) 0.239	0.84 (0.58, 1.22) 0.376
5	0.78 (0.51, 1.21) 0.277	0.75 (0.48, 1.16) 0.198	0.76 (0.48, 1.21) 0.260
≥6	0.64 (0.43,0.94) 0.026	0.59 (0.39, 0.90) 0.017	0.64 (0.43, 0.96) 0.039
*P* for trend	0.043	0.013	0.023

Model 1 was adjusted for none; Model 2 was adjusted for age and race; Model 3 was adjusted for age, race, education level, PIR, BMI, marital status, smoking status, alcohol intake, age at menarche, pelvic infection, ever use of female hormones, menstrual regularity, physical activity, depression, hypertension, diabetes, and hyperlipidemia. BMI, body mass index; DI-GM, dietary index for gut microbiota; OR, odds ratio; CI, confidence interval; PIR, poverty income ratio.

### 3.3 Sensitivity analysis

**Figure 3:**
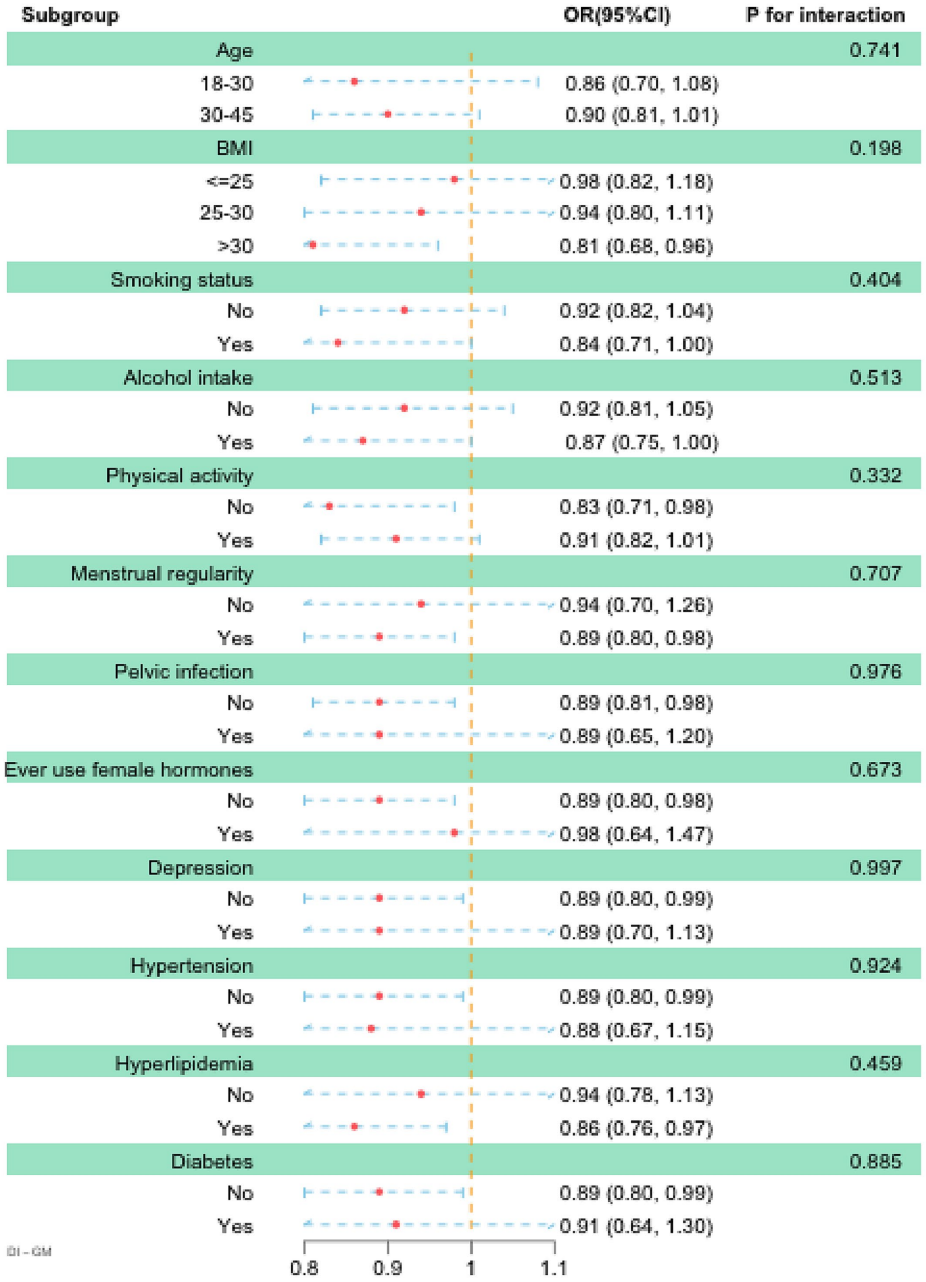
Subgroup analysis between DI-GM and infertility. Age, race, education level, PIR, BMI, marital status, smoking status, alcohol intake, age at menarche, pelvic infection, ever use of female hormones, menstrual regularity, physical activity, depression, hypertension, diabetes, and hyperlipidemia were adjusted. BMI, body mass index; DI-GM, dietary index for gut microbiota; PIR, poverty income ratio.

We conducted subgroup analyses stratified by age, BMI, smoking status, alcohol intake, physical activity, pelvic infection, ever use of female hormones, menstrual regularity, depression, hypertension, hyperlipidemia, and diabetes. The results of the subgroup analysis indicated that DI-GM was inversely associated with infertility (OR < 1) across all the examined subgroups, supporting its applicability and robustness across diverse populations. Moreover, although the *p*-values for interaction were not statistically significant in any of the subgroups, higher DI-GM scores were significantly and inversely associated with the prevalence of infertility among individuals with BMI > 30, inactive physical activity, regular menstruation, no history of pelvic infection, no use of female hormones, no depression, no hypertension, presence of hyperlipidemia, and absence of diabetes (Figure 3). Moreover, the results from multiple imputation and unweighted association analyses confirmed that the association between DI-GM and infertility remained significant and robust (Supplementary Tables S2–S3).

### 3.4 Mediation analysis

**Figure 4:**
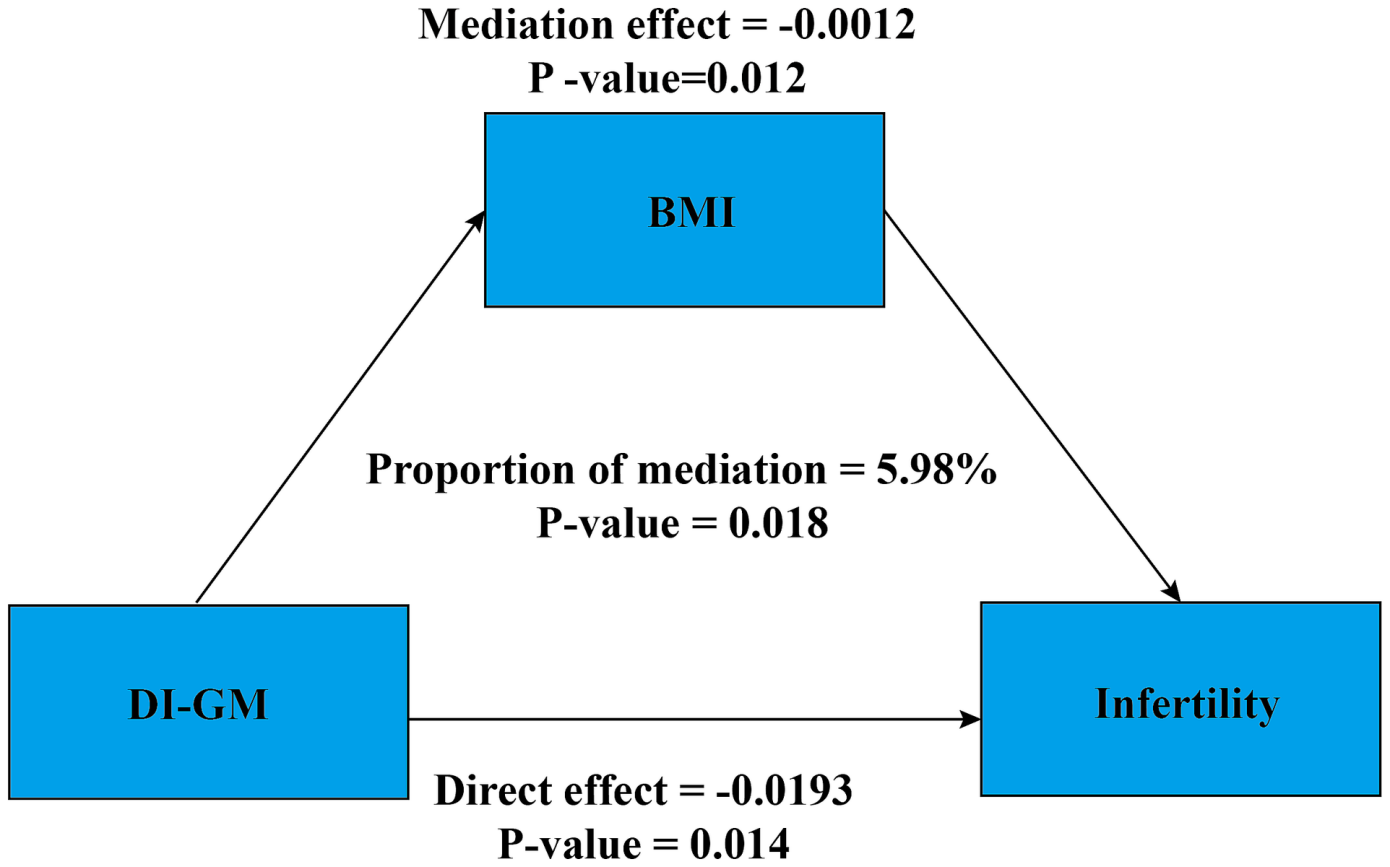
Mediation analysis. Age, race, education level, PIR, BMI, marital status, smoking status, alcohol intake, age at menarche, pelvic infection, ever use of female hormones, menstrual regularity, physical activity, depression, hypertension, diabetes, and hyperlipidemia were adjusted. BMI, body mass index; DI-GM, dietary index for gut microbiota; PIR, poverty income ratio.

Additionally, a mediation analysis was conducted to explore the potential mediating role of BMI in the association between DI-GM and infertility. After adjusting for all covariates, BMI exhibited a significant mediating effect on the association between DI-GM and infertility (Figure 4). The total effect coefficient of DI-GM on infertility mediated by BMI was −0.0206 (*p* = 0.006). The mediation effect was −0.0012 (*p* = 0.012). The direct effect was −0.019 (*p* = 0.014). The proportion of mediation was 5.98% (*p* = 0.018).

### 3.5 Establishment of the predictive nomogram

**Figure 5:**
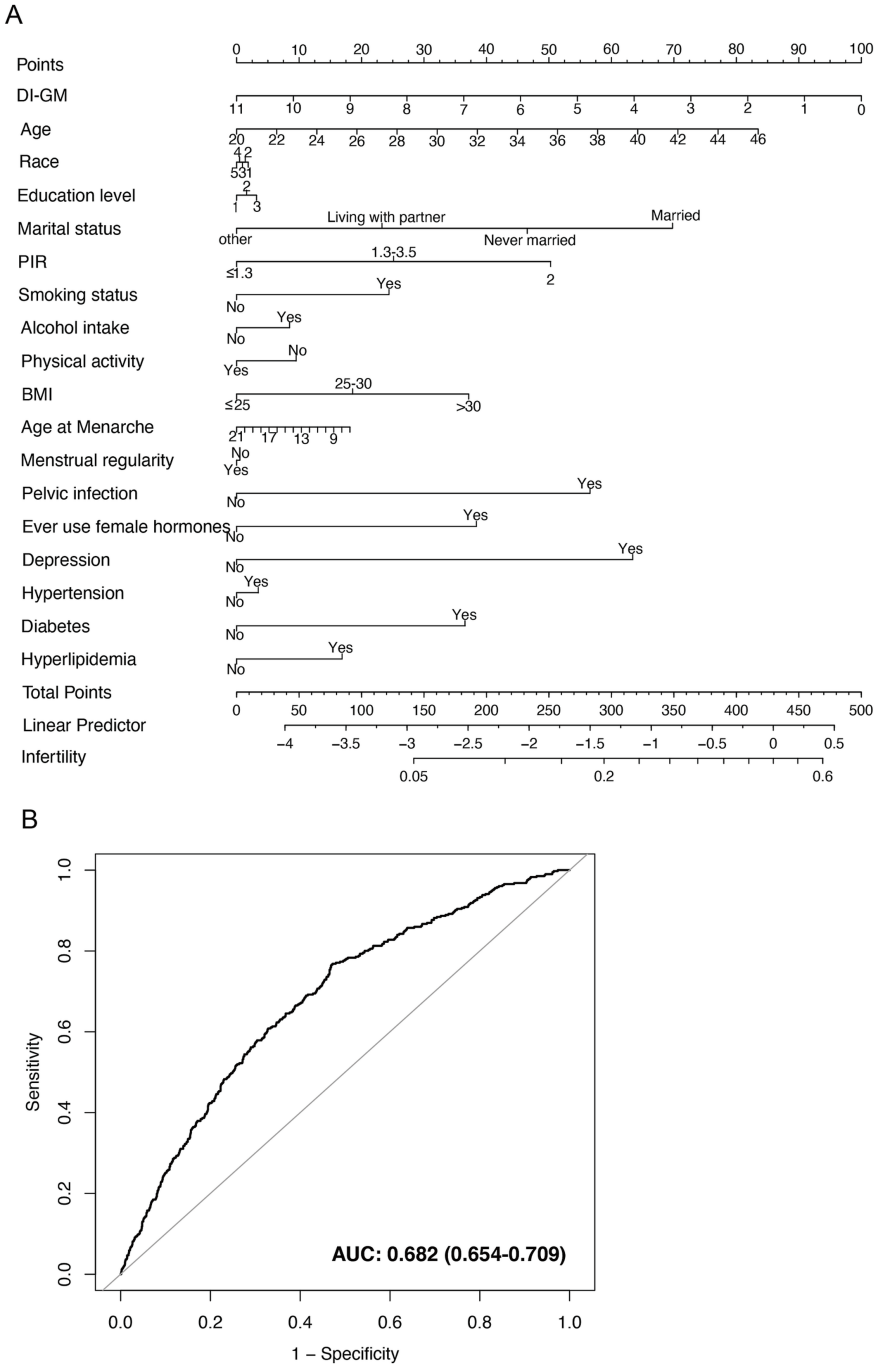
Nomogram for infertility risk prediction and its performance evaluation via the ROC curve. **(A)** Nomogram model based on Model 3. The numbers in the figure represent the following values: Race: 1 = Mexican American, 2 = Other Hispanic, 3 = Non-Hispanic White, 4 = Non-Hispanic Black, 5 = Other Race. Education level: 1 = Less than high school, 2 = High school or equivalent, 3 = College or above. **(B)** ROC curve based on Model 3, evaluating the predictive ability of the nomogram for infertility. BMI, body mass index; DI-GM, dietary index for gut microbiota; PIR, poverty income ratio.

In the fully adjusted model (Model 3), a nomogram was developed for the participants. In Model 3, each covariate was used as a predictor, with each row representing an individual predictor, allowing participants to select the corresponding value for each factor. Each predictor was assigned a specific score on a rating scale, and the total score was calculated by summing the points for all selected variables. A vertical line was then drawn downward from the total points to determine the corresponding probability of infertility. Higher scores were associated with an increased likelihood of infertility (Figure 5A). The accuracy of this nomogram in predicting outcomes was evaluated via a receiver operating characteristic (ROC) curve, which revealed an area under the curve (AUC) of 0.682 (95% CI: 0.654–0.709) (Figure 5B).

## 4 Discussion

To the best of our knowledge, this study constitutes the largest and first cross-sectional investigation exploring the relationship between the DI-GM and infertility. After participants were stratified on the basis of infertility status, multivariable-adjusted generalized linear models indicated that individuals with a DI-GM score ≥6 had a significantly lower risk of infertility than those with a score of 0–3 (OR = 0.64, 95% CI: 0.43–0.96). A smooth curve fitting analysis further illustrated this inverse association trend. Moreover, it is important to emphasize that the inverse association between the DI-GM score and infertility is not only statistically significant but also has meaningful clinical implications. After adjusting for potential confounders, individuals with a DI-GM score ≥6 presented a 36% lower risk of infertility than did those with a score of 0–3 (OR = 0.64, 95% CI: 0.43–0.96), suggesting that improving dietary patterns to increase DI-GM scores may serve as an effective strategy to reduce the prevalence of infertility. Although the odds ratio did not reach a very high magnitude, given the multifactorial nature of infertility, the observation that a single lifestyle factor can contribute to such a reduction in risk highlights its potential value as an intervention target, particularly in the context of primary healthcare and public health strategies. Sensitivity analyses confirmed the robustness and reliability of the findings. Notably, this study innovatively employed a mediation analysis model and revealed that BMI accounted for 5.98% of the mediating effect in the “DI-GM–infertility” association, providing novel evidence for elucidating the underlying pathophysiological mechanisms of this relationship.

Female infertility is a multifactorial condition influenced by genetic, environmental, and lifestyle factors (2, 23). Although the team led by Kase et al. (9) identified 14 gut microbiota-associated foods/nutrients and developed DI-GM scores through a systematic review, the precise relationship between DI-GM and infertility remains unclear. A study by Komiya et al. (35). revealed that beneficial effects on the gut microbiota were significantly reduced in infertile patients (4), suggesting that gut dysbiosis may contribute to the development of infertility. Previous studies have demonstrated a significant positive association between a healthy dietary pattern and live birth rates (2). The gut microbiota can respond rapidly to dietary changes and influence human health through multiple pathways (24). Therefore, consuming foods beneficial to the gut microbiota has emerged as a crucial strategy for maintaining overall health.

Numerous studies have demonstrated that foods beneficial to the gut microbiota can significantly increase fertility. A study by Gaskins et al. (5) revealed that a diet rich in fruits, vegetables, vitamins, and whole grains effectively improved embryo implantation rates, clinical pregnancy rates, and live birth rates. Vujkovic et al. (25) confirmed that adherence to a Mediterranean diet increased the probability of pregnancy by 40% (95% CI: 1.0–1.9). Similarly, Twigt et al. (26) reported that adherence to a Dutch healthy diet increased fertility by 65% (95% CI: 1.08–2.52). The gut microbiota may influence female reproductive function through the “gut–brain–reproductive axis.” Studies have shown that the gut microbiota can regulate the hypothalamic–pituitary–ovarian (HPO) axis via metabolic products such as short-chain fatty acids (SCFAs), thereby influencing the production of gonadotropin-releasing hormone (GnRH), follicle–stimulating hormone (FSH), and luteinizing hormone (LH). When gut dysbiosis occurs, these hormone levels may become dysregulated, leading to disruptions in estrogen secretion and follicular development, ultimately increasing the risk of infertility (4). Furthermore, gut dysbiosis may influence the vaginal microbiota composition through the “gut–vagina axis,” thereby affecting fertility (4). Probiotics and their metabolites can reshape the gut microbiota composition (27) and alleviate inflammation primarily through the production of short-chain fatty acids (SCFAs). The main SCFAs include acetate (AA), propionate (PA), butyrate (BA), and valerate (VA), which possess anti-inflammatory properties, help maintain intestinal mucosal barrier integrity, and increase insulin sensitivity (28). By binding to G protein-coupled receptors, SCFAs can ameliorate both systemic and local (intestinal) inflammation and metabolic dysfunction (29), thereby increasing fertility (30). Yang et al. (31) study revealed that avocado consumption significantly increased gut microbiota diversity, which has been closely linked to fertility levels (23). Our study revealed a significant inverse association between DI-GM scores and the prevalence of infertility. These findings, along with those of previous studies, provide strong support for the crucial role of the gut microbiota in regulating female fertility.

This study is the first to identify a significant mediating role of BMI in the relationship between DI-GM and infertility. These findings suggest that BMI serves as a partial mediator in the association between DI-GM and female infertility. This finding indicates that BMI may act as a potential physiological pathway involved in this association, providing insights into the mechanisms by which higher DI-GM scores are associated with a lower prevalence of infertility and offering a direction for future large-scale studies to explore this pathway further. This finding has important pathophysiological implications. First, obesity has been established as an independent risk factor for the onset and progression of infertility (7). Second, unhealthy dietary habits can lead to gut microbiota dysbiosis, promoting obesity and reducing fertility (32). A diet with a high DI-GM score can increase gut microbiota diversity, thereby increasing the likelihood of conception (8, 25, 26, 30). A high DI-GM score diet may regulate the gut microbiota balance, estrogen production, or butyrate generation (7), thereby improving BMI.

The strengths of this study include the use of a large, nationally representative NHANES dataset, which enhances the generalizability and credibility of the findings. We adjusted for multiple potential confounders to minimize bias and conducted sensitivity analyses to confirm the robustness of our results. However, several limitations remain. First, the cross-sectional design precludes the establishment of causal relationships. Longitudinal studies and randomized controlled trials are needed to confirm causality. Second, both dietary intake data and infertility diagnoses were self-reported, which may have introduced recall bias and misclassification bias. Future studies should incorporate more objective methods for dietary assessment and infertility diagnosis. Third, unmeasured confounders, such as genetic factors and direct gut microbiota sequencing data, may influence the results. Fourth, this study examined only BMI as a mediating variable, and future research should consider incorporating additional potential mediators, such as metabolic markers, inflammatory biomarkers, and hormone levels, to more comprehensively elucidate the underlying biological mechanisms linking DI-GM to female infertility. Fifth, some studies suggest that extreme dietary patterns may have detrimental effects on reproductive function. However, in the present study, the number of participants with extremely high DI-GM scores was very limited, precluding a robust statistical assessment of the association between extreme DI-GM values and infertility. Future studies with larger sample sizes encompassing a broader distribution of dietary patterns are warranted, which will help to further investigate the potential impact of extreme dietary behaviors on reproductive health.

This study has significant translational implications. In clinical settings, the DI-GM score could function as a primary screening tool for infertility risk stratification, enabling personalized dietary interventions for individuals with low DI-GM scores. Additionally, in this study, we developed an infertility risk prediction model based on the DI-GM and related covariates, which yielded an AUC of 0.682, indicating a moderate discriminative ability. Although the predictive accuracy has not reached a high level, the model’s performance is comparable to that of similar models reported in previous literature. For example, a prospective study involving 4,133 North American women attempting conception reported infertility prediction models using various algorithms, with AUCs ranging from 0.654 to 0.709, also reflecting moderate discriminative power (33). Another model based on demographic and clinical indicators yielded an AUC of 0.60 (34). Therefore, the model developed in our study demonstrates a certain degree of comparability and reference value in terms of predictive performance. More importantly, this study is the first to incorporate DI-GM into an infertility risk prediction model, highlighting its modifiability and potential for broad application, as DI-GM is a dietary-based, intervention-targetable metric. The use of a nomogram as a visual tool enables clinicians to perform individualized risk assessments on the basis of easily obtainable parameters such as dietary patterns, BMI, lifestyle factors, and menstrual characteristics. For individuals at high risk, early identification and targeted interventions—such as dietary modifications and lifestyle optimization—may help reduce the risk of infertility. Therefore, despite the model’s moderate AUC value, its potential utility in public health management, nutritional interventions, and clinical risk stratification should not be overlooked. Further validation and promotion in larger and longitudinal studies are warranted.

In conclusion, this study is the first to reveal an inverse association between DI-GM scores and infertility prevalence. These findings not only provide new evidence supporting the “diet–gut microbiota–infertility” pathway but also suggest that optimizing dietary patterns improves the gut microbiota composition. However, given the cross-sectional nature of this study, causal relationships remain to be established. Future longitudinal studies are warranted to further explore the potential role of DI-GM in the prevention and intervention of infertility.

## Data Availability

The original contributions presented in the study are included in the article/Supplementary material, further inquiries can be directed to the corresponding author.
