# Supplementary material for: Association between dietary index for gut microbiota and female infertility: a cross-sectional NHANES 2013–2020
Source: Front Nutr. 2025 Apr 23;12:1587240. doi: 10.3389/fnut.2025.1587240 (PMC12055548; doi:10.3389/fnut.2025.1587240)
Supplement: Supplementary file 1 [file Table_1.docx]

**Supplementary Materials Files**

To: **Association between Dietary Index for Gut Microbiota and female infertility: a cross-sectional NHANES 2013--2020**

by Yu Fu, Mengling Peng, Bing Li, Yaoting Zhang, Yang Zheng

**Supplementary Method**

***Multiple imputation***

**Multiple imputation is a robust and widely used statistical technique for handling missing data across various fields, including epidemiology, social sciences, and clinical research. The method involves generating several plausible datasets through imputation, conducting separate analyses on each dataset, and pooling the results to produce valid estimates and measures of uncertainty^1,2^. This technique helps mitigate potential biases and ensures the representativeness of the findings.**

**The multiple imputation by chained equations (MICE) method, employed in this study, is a flexible approach that sequentially imputes missing values for each variable by developing regression models, conditioned on the observed values of other variables^1,2^. We created five imputed datasets and combined the results according to Rubin’s Rules, ensuring the robustness and reliability of our conclusions.**

**References:**

1. Beesley LJ, Bondarenko I, Elliot MR, Kurian AW, Katz SJ, Taylor JM. Multiple imputation with missing data indicators. *Stat Methods Med Res*. Dec 2021;30(12):2685-2700. doi:10.1177/09622802211047346

2. White IR, Royston P, Wood AM. Multiple imputation using chained equations: Issues and guidance for practice. *Stat Med*. Feb 20 2011;30(4):377-99. doi:10.1002/sim.4067

**Supplementary Table:**

**Table S1. Components and scoring criteria of DI-GM in NHANES.**

**Table S2. Association between DI-GM and infertility of the NHANES 2013-2020 participants after** **multiple imputation**

**Table S3. Unweighted association between DI-GM and infertility of the NHANES 2013-2020 participants**

**Table S4 STROBE Statement Checklist**

**Table S1. Components and scoring criteria of DI-GM in NHANES.**

| **Components of DI-GM** | **Food items included in NHANES** | **Scoring criteria** |
| --- | --- | --- |
| **Beneficial to gut microbiota** | Avocados | Score 1 - Consumption≥sex-specific median  Score 0 - Otherwise |
|  | Broccoli |  |
|  | Chickpeas |  |
|  | Coffee |  |
|  | Cranberries |  |
|  | Fermented dairy (including yogurt, cheese, kefir, sour cream, buttermilk) |  |
|  | Fiber  Green Tea |  |
|  | Soybean (including Soy milk, Tofu) |  |
|  | Whole grains |  |
| **Unfavorable to gut microbiota** | Refined grains | Score 0 - Consumption≥sex-specific median  Score 1 - Otherwise |
|  | Processed meat |  |
|  | Red meat |  |
|  | High-fat diet (% energy) | Score 0 - Consumption≥40%  Score 1 - Otherwise |

Abbreviations: DI-GM, dietary index for gut microbiota; NHANES, National Health and Nutrition Examination Survey.

**Table S2. Association between DI-GM and infertility of the NHANES 2013-2020 participants after** **multiple imputation**

| Variable | Model1 | Model2 | Model3 |
| --- | --- | --- | --- |
|  | OR (95% CI) P-value | OR (95% CI) P-value | OR (95% CI) P-value |
| DI-GM | 0.90 (0.83, 0.98) 0.019 | 0.88 (0.80, 0.96) 0.006 | 0.88 (0.81, 0.97) 0.009 |
| DI-GM group |  |  |  |
| 0-3 | Reference | Reference | Reference |
| 4 | 0.82 (0.59, 1.15) 0.257 | 0.79 (0.56, 1.10) 0.162 | 0.84 (0.62, 1.16) 0.299 |
| 5 | 0.71 (0.48, 1.06) 0.103 | 0.68 (0.45, 1.02) 0.068 | 0.69 (0.45, 1.06) 0.099 |
| ≥6 | 0.65 (0.46,0.91) 0.017 | 0.58 (0.40, 0.85) 0.007 | 0.60 (0.42, 0.86) 0.010 |
| P for trend | 0.001 | 0.004 | 0.006 |

Model: 1 adjusted for none; Model 2: adjusted for age and race; Model 3: adjusted for age, race, education level, PIR, BMI, marital status, smoking status, alcohol intake, physical activity, age at menarche, pelvic infection, ever use of female hormones, menstrual regularity, depression, hypertension, diabetes, and hyperlipidemia. Abbreviations: BMI: body mass index; DI-GM: dietary index for gut microbiota; OR, odd ratio; CI, confidence interval; PIR: poverty income ratio

**Table S3. Unweighted association between DI-GM and infertility of the NHANES 2013-2020 participants**

| Variable | Model1 | Model2 | Model3 |
| --- | --- | --- | --- |
|  | OR (95% CI) P-value | OR (95% CI) P-value | OR (95% CI) P-value |
| DI-GM | 0.93 (0.87, 0.99) 0.026 | 0.91 (0.85, 0.98) 0.008 | 0.91 (0.85, 0.98) 0.015 |
| DI-GM group |  |  |  |
| 0-3 | Reference | Reference | Reference |
| 4 | 0.67 (0.51, 0.90) 0.007 | 0.65 (0.48, 0.86) 0.003 | 0.69 (0.51, 0.93) 0.015 |
| 5 | 0.84 (0.63, 1.12) 0.239 | 0.79 (0.59, 1.06) 0.120 | 0.82 (0.61, 1.10) 0.187 |
| ≥6 | 0.69 (0.51,0.92) 0.013 | 0.64 (0.47, 0.86) 0.003 | 0.66 (0.48, 0.90) 0.009 |
| P for trend | 0.044 | 0.012 | 0.017 |

Model: 1 adjusted for none; Model 2: adjusted for age and race; Model 3: adjusted for *age, race, education level, PIR, BMI, marital status, smoking status, alcohol intake, physical activity, age at menarche, pelvic infection, ever use of female hormones, menstrual regularity, depression, hypertension, diabetes, and hyperlipidemia*. Abbreviations: BMI: body mass index; DI-GM: dietary index for gut microbiota; OR, odd ratio; CI, confidence interval; PIR: poverty income ratio.

**Table S4 STROBE Statement Checklist**

**Item**

**No Recommendation**

**Page No**

| **Title and abstract** 1 (*a*) Indicate the study’s design with a commonly used term in the title or the  abstract  (*b*) Provide in the abstract an informative and balanced summary of what was done and what was found | 1-2  1-2 |
| --- | --- |

**Introduction**

| Background/rationale | 2 | Explain the scientific background and rationale for the investigation being reported | 2 |
| --- | --- | --- | --- |
| Objectives | 3 | State specific objectives, including any prespecified hypotheses | 2 |

| **Methods**  Study design | 4 Present key elements of study design early in the paper | | 5 | |  |
| --- | --- | --- | --- | --- | --- |
| Setting | 5 | Describe the setting, locations, and relevant dates, including periods of recruitment, exposure, follow-up, and data collection | 5 | |  |
| Participants | 6 | (*a*) Give the eligibility criteria, and the sources and methods of selection of participants. Describe methods of follow-up  (*b*) For matched studies, give matching criteria and number of exposed and unexposed | 5 | |  |
| Variables | 7 | Clearly define all outcomes, exposures, predictors, potential confounders, and effect modifiers. Give diagnostic criteria, if applicable | 6-7 | |  |
| Data sources/ measurement | 8* | For each variable of interest, give sources of data and details of methods of assessment (measurement). Describe comparability of assessment methods if there is more than one group | 6-7 | |  |
| Bias | 9 | Describe any efforts to address potential sources of bias | NA | |  |
| Study size | 10 | Explain how the study size was arrived at | NA | |  |
| Quantitative variables | 11 | Explain how quantitative variables were handled in the analyses. If applicable, describe which groupings were chosen and why | 5 | |  |
| Statistical methods | 12 | (*a*) Describe all statistical methods, including those used to control for confounding  (*b*) Describe any methods used to examine subgroups and interactions  (*c*) Explain how missing data were addressed  (*d*) If applicable, explain how loss to follow-up was addressed (*e*) Describe any sensitivity analyses | 6-7  6-7  8 | |  |
| **Results** |  |  |  | |  |
| Participants | 13* | (a) Report numbers of individuals at each stage of study—eg numbers potentially eligible, examined for eligibility, confirmed eligible, included in the study,  completing follow-up, and analysed  (b) Give reasons for non-participation at each stage  (c) Consider use of a flow diagram | 5  5  5 | |  |
| Descriptive data | 14* | (a) Give characteristics of study participants (eg demographic, clinical, social) and information on exposures and potential confounders  (b) Indicate number of participants with missing data for each variable of interest | 8  5 | |  |
| (c) Summarise follow-up time (eg, average and total amount) | | | | NA | |
| Outcome data 15* Report numbers of outcome events or summary measures overtime | | | | 6 | |

| Main results | 16 | (*a*) Give unadjusted estimates and, if applicable, confounder-adjusted estimates and their precision (eg, 95% confidence interval). Make clear which confounders were adjusted for and why they were included  (*b*) Report category boundaries when continuous variables were categorized  (*c*) If relevant, consider translating estimates of relative risk into absolute risk for a meaningful time period | 9-10  9-10 |
| --- | --- | --- | --- |
| Other analyses | 17 | Report other analyses done—eg analyses of subgroups and interactions, and sensitivity analyses | 9-10 |
| **Discussion** | | | |
| Key results | 18 | Summarise key results with reference to study objectives | 13 |
| Limitations | 19 | Discuss limitations of the study, taking into account sources of potential bias or imprecision. Discuss both direction and magnitude of any potential bias | 15 |
| Interpretation | 20 | Give a cautious overall interpretation of results considering objectives, limitations, multiplicity of analyses, results from similar studies, and other relevant evidence | 13-16 |
| Generalisability | 21 | Discuss the generalisability (external validity) of the study results | 13-16 |
| **Other information** | | | |
| Funding | 22 | Give the source of funding and the role of the funders for the present study and, if applicable, for the original study on which the present article is based | No  funding |

*Give information separately for exposed and unexposed groups.

**Note:** An Explanation and Elaboration article discusses each checklist item and gives methodological background and published examples of transparent reporting.
